# A universal testing and treatment intervention to improve HIV control: One-year results from intervention communities in Zambia in the HPTN 071 (PopART) cluster-randomised trial

**DOI:** 10.1371/journal.pmed.1002292

**Published:** 2017-05-02

**Authors:** Richard Hayes, Sian Floyd, Ab Schaap, Kwame Shanaube, Peter Bock, Kalpana Sabapathy, Sam Griffith, Deborah Donnell, Estelle Piwowar-Manning, Wafaa El-Sadr, Nulda Beyers, Helen Ayles, Sarah Fidler, for the HPTN 071 (PopART) Study Team

**Affiliations:** 1 Department of Infectious Disease Epidemiology, London School of Hygiene & Tropical Medicine, London, United Kingdom; 2 Zambart, University of Zambia School of Medicine, Lusaka, Zambia; 3 Desmond Tutu TB Centre, Department of Paediatrics and Child Health, University of Stellenbosch, Stellenbosch, South Africa; 4 FHI 360, HIV Prevention Trials Network, Durham, North Carolina, United States of America; 5 HIV Prevention Trials Network Statistical and Data Management Center, Statistical Center for HIV/AIDS Research and Prevention, Seattle, Washington, United States of America; 6 HIV Prevention Trials Network Laboratory Center, Johns Hopkins University School of Medicine, Baltimore, Maryland, United States of America; 7 Mailman School of Public Health, Columbia University, New York, New York, United States of America; 8 Department of Clinical Research, London School of Hygiene & Tropical Medicine, London, United Kingdom; 9 HIV Clinical Trials Unit, Imperial College London, London, United Kingdom; University of Bern, SWITZERLAND

## Abstract

**Background:**

The Joint United Nations Programme on HIV/AIDS (UNAIDS) 90-90-90 targets require that, by 2020, 90% of those living with HIV know their status, 90% of known HIV-positive individuals receive sustained antiretroviral therapy (ART), and 90% of individuals on ART have durable viral suppression. The HPTN 071 (PopART) trial is measuring the impact of a universal testing and treatment intervention on population-level HIV incidence in 21 urban communities in Zambia and South Africa. We report observational data from four communities in Zambia to assess progress towards the UNAIDS targets after 1 y of the PopART intervention.

**Methods and findings:**

The PopART intervention comprises annual rounds of home-based HIV testing delivered by community HIV-care providers (CHiPs) who also support linkage to care, ART retention, and other services. Data from four communities in Zambia receiving the full intervention (including immediate ART for all individuals with HIV) were used to determine proportions of participants who knew their HIV status after the CHiP visit; proportions linking to care and initiating ART following referral; and overall proportions of HIV-infected individuals who knew their status (first 90 target) and the proportion of these on ART (second 90 target), pre- and post-intervention. We are not able to assess progress towards the third 90 target at this stage of the study. Overall, 121,130 adults (59,283 men and 61,847 women) were enumerated in 46,714 households during the first annual round (December 2013 to June 2015). Of the 45,399 (77%) men and 55,703 (90%) women consenting to the intervention, 80% of men and 85% of women knew their HIV status after the CHiP visit. Of 6,197 HIV-positive adults referred by CHiPs, 42% (95% CI: 40%–43%) initiated ART within 6 mo and 53% (95% CI: 52%–55%) within 12 mo. In the entire population, the estimated proportion of HIV-positive adults who knew their status increased from 52% to 78% for men and from 56% to 87% for women. The estimated proportion of known HIV-positive individuals on ART increased overall from 54% after the CHiP visit to 74% by the end of the round for men and from 53% to 73% for women. The estimated overall proportion of HIV-positive adults on ART, irrespective of whether they knew their status, increased from 44% to 61%, compared with the 81% target (the product of the first two 90 targets). Coverage was lower among young men and women than in older age groups. The main limitation of the study was the need for assumptions concerning knowledge of HIV status and ART coverage among adults not consenting to the intervention or HIV testing, although our conclusions were robust in sensitivity analyses.

**Conclusions:**

In this analysis, acceptance of HIV testing among those consenting to the intervention was high, although linkage to care and ART initiation took longer than expected. Knowledge of HIV-positive status increased steeply after 1 y, almost attaining the first 90 target in women and approaching it in men. The second 90 target was more challenging, with approximately three-quarters of known HIV-positive individuals on ART by the end of the annual round. Achieving higher test uptake in men and more rapid linkage to care will be key objectives during the second annual round of the intervention.

**Trial registration:**

ClinicalTrials.gov NCT01900977

## Abstract 2

Richard Hayes and colleagues report data from a large HIV testing and treatment trial with implications for HIV control in Africa.

## Author summary

**Why was this study done?:**

- Epidemiological and modelling studies have shown that universal testing and treatment (UTT) for HIV, whereby everyone in the community should know their HIV status and all HIV-infected individuals should be offered immediate antiretroviral therapy (ART), may steeply reduce the incidence of new HIV infections in generalised epidemics in sub-Saharan Africa.
- However, it is not known if such UTT interventions can be implemented on a large scale in resource-poor settings, or what coverage can be achieved in practice.

**What did the researchers do and find?:**

- We are carrying out a large community-randomised trial, the HPTN 071 (PopART) trial, to measure the impact of a UTT intervention on HIV incidence at the population level over three years in 21 urban communities in Zambia and South Africa.
- We report here on the uptake of the intervention achieved in four Zambian communities after the first annual round of the intervention, which is delivered house-to-house by trained lay health workers.
- Uptake of HIV testing with the lay health workers was high, and we found that after one year, an estimated 78% of men and 87% women who were HIV-positive knew their HIV status. Among known HIV-positive adults, around 73% were on ART by the end of the annual round.
- The estimated overall proportion of HIV-positive adults on ART, irrespective of whether they knew their status, increased from 44% to 61% following the intervention.
- The main challenges were that many men could not be contacted during home visits, that linkage to care and initiation of ART were slower than anticipated, and that overall coverage was lower in young people.

**What do these findings mean?:**

- The Joint United Nations Programme on HIV/AIDS (UNAIDS) 90-90-90 targets require that, by 2020, 90% of HIV-positive individuals know their status (first 90 target), 90% of known HIV-positive individuals are on ART (second 90 target), and 90% of those have durable viral suppression (third 90 target).
- We have shown that after the first annual round of a community-wide household-based intervention, knowledge of HIV-positive status was close to the first 90 target in women, and approaching it in men. There was a substantial increase in the proportion of known HIV-positive individuals on ART, although the second 90 target has not yet been reached.
- The repeated annual rounds of household visits are a strength of the PopART intervention, and, based on the findings of the current analysis, future rounds will focus on key challenges, including reaching men and young people and achieving more rapid linkage to care and ART initiation.

## Introduction

While there have been some reductions globally in the incidence of new HIV infections, incidence and prevalence remain at unacceptably high levels in many parts of sub-Saharan Africa. This is especially the case in southern Africa, where many regions are still experiencing severe generalised epidemics. Thanks to the large-scale rollout of antiretroviral therapy (ART), mortality from HIV has fallen [1]. HIV incidence has not fallen to the same extent, however, and the number of new HIV infections each year exceeds the number of deaths, meaning that the total number of HIV-infected individuals continues to increase [1]. Unless effective ways can be found of steeply reducing incidence, it will become increasingly difficult to sustain ART services for all who need them.

Wide-scale provision of ART is now recognised as a key preventive intervention for HIV control. The concept of treatment as prevention is based on evidence showing that HIV transmission is strongly correlated with viral load, and that the risk of transmission is very low if virus is undetectable [2–5]. The HPTN 052 trial showed definitively that early initiation of ART reduced HIV transmission by an estimated 96% [3]. Mathematical modelling has shown that if a large enough proportion of the HIV-positive population is diagnosed and started on treatment, with good enough retention and adherence to achieve high rates of viral suppression, this could lead to steep reductions in HIV incidence and potentially to the long-term elimination of HIV as a public health problem [6–9]. Observational data from a range of settings have demonstrated correlations between ART coverage at the population level and reductions in HIV incidence [10–14].

Based on this evidence, the Joint United Nations Programme on HIV/AIDS (UNAIDS) has promulgated a set of targets aimed at ensuring that a high proportion of the HIV-infected population is diagnosed, on ART, and virally suppressed, with the aim of reducing HIV incidence as well as protecting the health of HIV-infected individuals [15,16]. The 90-90-90 targets are to be achieved by 2020 and require that 90% of all people living with HIV know their status, that 90% of all people diagnosed with HIV receive sustained ART, and that 90% of all people receiving ART have durable viral suppression [15]. Taken together, meeting these targets would imply that approximately 73% of HIV-infected individuals would be virally suppressed. An even more ambitious set of 95-95-95 targets is to be achieved by 2030, with the aim of substantially reducing HIV incidence and prevalence by then [16].

While it may be useful to have aspirational targets for service coverage, it is not known whether such targets can be achieved on a large scale at the population level, or what are the most effective and cost-effective methods of achieving them. Changes to WHO guidelines to recommend immediate initiation of ART for all people living with HIV, irrespective of CD4 count, will remove one barrier to achieving the second 90 target. However, there are many other obstacles and challenges to ensuring universal testing and treatment (UTT), including the need to scale up HIV testing in the community so that everyone knows their current HIV status, and the need to ensure that all HIV-infected individuals are linked to care and commence ART rapidly, with high rates of retention, adherence, and viral suppression.

The HPTN 071 (PopART) trial is implementing a combination prevention programme in Zambia and South Africa aiming to achieve UTT through a house-to-house service delivered by a cadre of community health workers, and to measure its effectiveness and cost-effectiveness in reducing HIV incidence at the population level [17]. In this paper, we examine findings from the trial communities receiving the PopART UTT intervention in Zambia after the first year of the intervention. We examine what coverage has been achieved following the first annual round, what difference this has made to key indicators of uptake, and how close we have come to the first two of the 90-90-90 targets, which are important process outcomes for the trial. We also ask what are the most important barriers to meeting these targets. The impact of the PopART intervention on the primary outcome (HIV incidence) will be reported following trial completion in 2018.

## Methods

### Trial design

**Fig 1:**
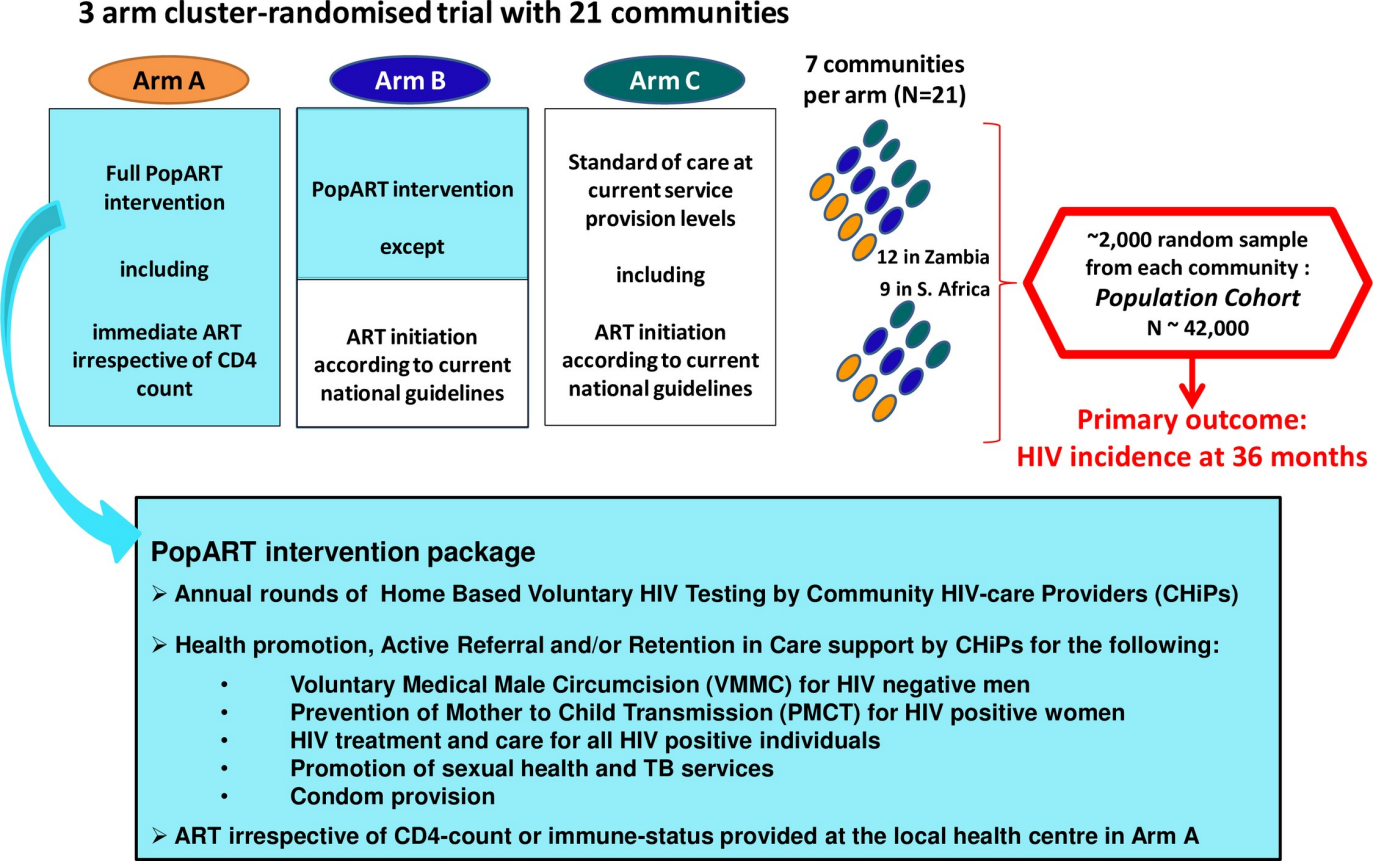
Overview of design of the HPTN 071 (PopART) trial. ART, antiretroviral therapy; TB, tuberculosis.

Full details of the HPTN 071 (PopART) trial and intervention have been presented previously [17]. In brief (Fig 1), 21 urban and peri-urban communities in Zambia and South Africa (each community defined as the catchment population of a government health facility at which services for HIV care and ART provision were already in place) were formed into seven matched triplets (four in Zambia and three in South Africa) and randomly allocated to three study arms. Arm A is receiving the full PopART intervention including immediate ART irrespective of CD4 count. Arm B is receiving the full intervention except that ART is provided according to current national treatment guidelines. Arm C is a control arm, which continues to receive existing standard of care including ART according to current national treatment guidelines.

To measure the impact of the intervention, a population cohort comprising a random sample of adults aged 18–44 y (approximately 2,000 per community) has been enrolled. Following a baseline survey, the cohort will be followed up at annual intervals for 3 y. The primary objective of the trial is to measure the population-level impact of the intervention by comparing HIV incidence over 36 mo between the three study arms.

Here we report data on the uptake and coverage of the UTT intervention after the first annual round of intervention in the entire population of our Arm A communities in Zambia. During the first annual round, some challenges were encountered in the collection of intervention data from our South African communities. In particular, there was some under-recording of community members who were absent or declined to participate in the intervention or who declined an HIV test. These problems have since been resolved, and we plan to report uptake and coverage in South Africa during later rounds of intervention in due course.

### Intervention

The PopART intervention is a combination prevention package with several components. A cadre of community HIV-care providers (CHiPs) provides house-to-house services for all members of the community, including annual HIV testing and counselling; linkage to care at the local government health facility and support for adherence and retention in care for those identified as HIV-positive; promotion of voluntary medical male circumcision (VMMC) for HIV-negative uncircumcised men and referral to local VMMC services; promotion of prevention of mother-to-child transmission (PMTCT) services for pregnant women; referral of those reporting symptoms of sexually transmitted infections for diagnosis and treatment at the health facility; screening for symptoms of tuberculosis (TB) and linkage to care for those diagnosed with TB; and promotion and provision of condoms. ART is provided as a routine service by the health facility.

CHiPs work in pairs, with each pair serving a zone consisting of around 500 households. They visit all households in their zone to provide annual testing and re-testing using HIV rapid tests, as well as making additional visits as necessary to complete intervention provision and follow-up. They record basic data on the household and all household members on an electronic data capture (EDC) device, with more detailed data (e.g., on HIV test history, ART status, and uptake of HIV testing and other services provided by CHiPs) recorded for adults aged 18 y and above who consent to receive the intervention. The EDC populates a CHiP database, which is used to obtain process measures on the uptake of the intervention. There is no fixed schedule for follow-up visits during the annual round, but special efforts are made to follow up all participants known to be HIV-positive to support them to link to and stay in HIV care, as well as to record information on these outcomes.

The PopART intervention will be delivered in four annual rounds. In this paper, we report the outcome of the first annual round (delivered from December 2013 to June 2015, with some variation as to when services were rolled out in communities) in the four Arm A communities in Zambia, to determine how close a UTT intervention can come to meeting the first two of the 90-90-90 targets after a single year of intervention. While services including testing and linkage to care are offered for all household members, ethical approval for data collection during the first annual round was obtained only for adults aged 18 y and over, and so analyses are restricted to this age group.

### Statistical methods

The statistical analysis plan for the parent HPTN 071 (PopART) study is currently under development and review, and had not been finalised at the time that the process data analysis reported in this paper was carried out. The indicators of intervention coverage used for the current analyses were determined at the start of the trial, but when the UNAIDS 90-90-90 targets were announced at a later stage (September 2014), our analyses were extended to provide estimates of coverage against these targets.

Data from the CHiP database were first used to populate a cascade of care diagram showing the proportions completing each step required if individuals are to know their HIV status and initiate ART. Separate estimates were obtained for men and women, and the data were pooled across all four communities in Arm A. First, we calculated the proportion of households visited during the first annual round, with the denominator (number of households) obtained from a census carried out prior to the trial, and, second, we calculated the proportion of visited households that consented both to the intervention being explained and to enumeration (listing) of all household members on the EDC device. Third, we calculated the proportion of enumerated adult members who were contacted and consented to take part in the PopART intervention and, of these, the proportion who knew their HIV status following the CHiP visit because they accepted the offer of an HIV test from the CHiP, self-reported as HIV-positive, or self-reported as HIV-negative based on a test within the previous 3 mo. Fourth, we computed the number and proportion of “known HIV-positive” adults who were referred to care by the CHiP, overall and with restriction to those who had never previously registered for HIV care. Finally, we computed the proportion of those referred who had linked to care and commenced ART by 6 mo and 12 mo after referral by a CHiP. Because individuals were referred at different times, with differing durations of follow-up, these proportions were estimated by Kaplan–Meier “time to event” analysis, using retrospective information from follow-up visits by CHiPs up to mid-2016 but with follow-up right-censored at the end of the first annual round (30 June 2015). The corresponding survival plots were obtained from the same analysis.

This cascade of care focuses on the success of the intervention in contacting adults, achieving high rates of participation among those contacted, achieving high rates of test uptake among those who consent to participate, and linking to care those HIV-positive adults who are not already on ART. We carried out further analyses to obtain a more complete picture of the overall proportion of adults who knew their HIV status, the overall proportion known to be HIV-positive, and the proportion of known HIV-positive adults who were in HIV care or on ART prior to the intervention, and to determine how much these proportions were increased by the intervention. Using the CHiP database, among those consenting to the PopART intervention, we computed the proportion who knew their HIV status (and the proportion known to be HIV-positive) among all adults, before and after the CHiP visit. Taking those who were known HIV-positive after the CHiP visit as the denominator, we then computed the proportions in care and on ART at the time of the CHiP visit, as well as at the end of the first annual round of intervention (using as the denominator those still resident in the same zone of the community according to the most recent follow-up information, based on follow-up data collected on or before 30 June 2015).

Finally, we used these data to estimate coverage against the first two of the 90-90-90 targets: proportion of individuals with knowledge of HIV-positive status and proportion of known HIV-positive individuals on ART. All calculations were stratified by sex, community, and age group. Full details of the computations of these estimates are given in S1–S4 Tables, illustrated using data for women in one age group. We present coverage estimates separately for men and women of all ages, and also by age group.

We first estimated coverage among all adults who consented to the PopART intervention. To estimate the proportion of HIV-positive individuals who knew their status before and after the CHiP visit, we assumed, first, that all those who knew they were HIV-positive self-reported this to the CHiP and, second, that among those whose HIV status was not known to the CHiP (because they declined to test with the CHiP and did not self-report that they were HIV-positive or that they had tested HIV-negative within the previous 3 mo), the HIV prevalence was the same as among those who accepted a test (Assumption A). Coverage against the first 90 target was estimated before and immediately after the CHiP visit, using data on HIV test uptake and results. Coverage against the second 90 target, based on those self-reporting that they were in HIV care or currently on ART among those known HIV-positive, was estimated before and immediately after the CHiP visit, and also at the end of the first annual round to take account of participants newly initiated on ART after the CHiP visit.

To estimate overall coverage against the first two 90 targets in the entire adult population of these communities, some additional assumptions were needed. This was because no data were available for households that did not consent to enumeration of household members and because, among households that consented to enumeration, the only information available for adults who did not consent to participate was their age and sex. We assumed, first, that HIV prevalence was the same in those consenting and not consenting to the intervention (Assumption B); second, that the proportion of HIV-positive individuals who knew their HIV status prior to the CHiP intervention was the same in those consenting and not consenting (Assumption C); and, third, that the proportions of known HIV-positive individuals in care or on ART prior to the CHiP intervention were the same in those consenting and not consenting (Assumption D). All of the above assumptions (A to D) were assumed to apply within strata defined by sex, community, and age group.

We conducted two further analyses to explore the sensitivity of the above coverage estimates to the assumptions made. First, for each assumption (A to D), we assumed that the true proportion was equal to the assumed value or was increased or decreased by a factor of 1.25. We examined the range of coverage estimates resulting from these 81 (3^4^) combinations of values, and provide the minimum and maximum values together with the central estimate. Second, we made alternative assumptions about adults whose HIV status was not known to CHiPs: these alternative assumptions were that HIV prevalence was twice as high as among those who tested with the CHiPs, that the percentage who knew their HIV-positive status was the same as in those whose HIV status was known to CHiPs, and that ART uptake among those who knew their HIV-positive status was either the same or half the value as among those who self-reported as HIV-positive to the CHiPs.

### Laboratory methods

CHiP participants were initially screened for HIV infection by fingerprick using the Alere Determine HIV-1/2 test (Alere International). Participants with a non-reactive test had no further testing. Participants with reactive results were further tested with the Uni-Gold HIV test (Trinity Biotech). If results were concordant, the participant was referred for care. If results were discordant, the testing was repeated. If the results were still discordant, the CHiPs would return 2 wk later for follow-up testing. If results were discordant again, a venous blood sample was collected and sent to the laboratory for further testing using the rapid tests and, if indicated, testing using the Abbott Architect HIV Ag/Ab Combo Assay. This test algorithm is in keeping with Zambian national guidelines.

### Ethical considerations

During CHiP visits to households, all adults were asked for verbal informed consent to take part in the PopART intervention, which included the collection of data on the CHiP EDC. Those agreeing to a rapid HIV test gave written consent according to standard health care procedures for HIV testing and counselling in Zambia. The study and all the above procedures were approved by the ethics committees of the London School of Hygiene & Tropical Medicine and the University of Zambia.

## Results

### Cascade of care

**Fig 2:**
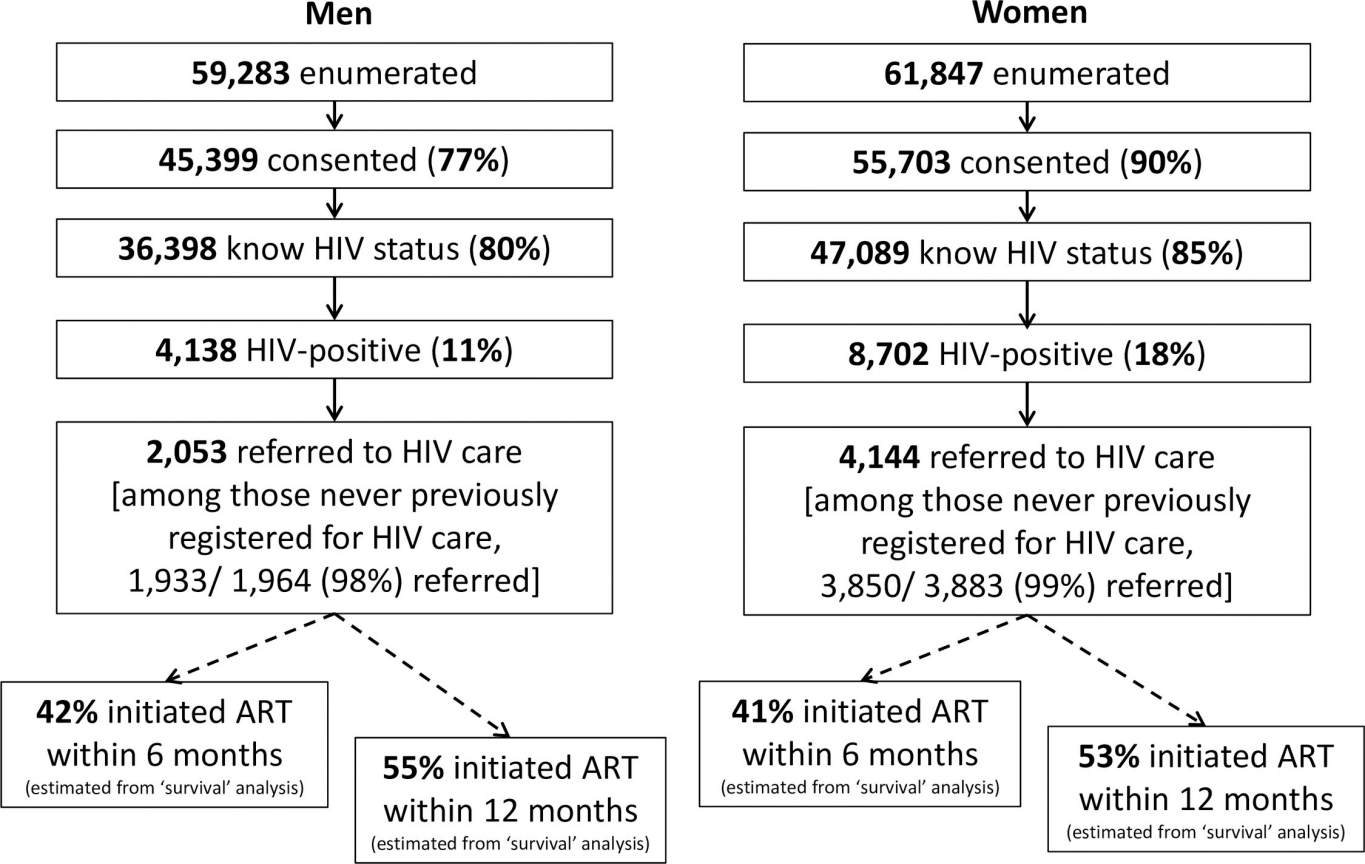
Cascade of care from enumeration of household members through ART initiation during the first annual round of the PopART intervention. ART, antiretroviral therapy.

A total of 48,583 households were visited during the first annual round in the four study communities in Zambia, close to 100% of all households listed during the community census prior to the study. Enumeration of individual household members was completed in 46,714 (96%) of the households visited, with a total of 121,130 adults aged 18 y and over (59,283 men and 61,847 women) (Fig 2). Of those enumerated, 77% of men and 90% of women consented to the PopART intervention. Of those consenting, 80% of men and 85% of women knew their HIV test status following the CHiP visit, because they self-reported that they were HIV-positive, because they accepted the offer of an HIV rapid test by the CHiP, or because they self-reported that they had tested HIV-negative in the previous 3 mo.

Following the CHiP visit, a total of 4,138 men and 8,702 women were known to be HIV-positive, representing 11% and 18% of those who knew their HIV status among men and women, respectively. Of these, 2,133 men and 4,765 women reported that they were already in HIV care, and 1,941 and 4,308, respectively, were on ART. Among those not on ART, CHiPs referred a total of 2,053 men and 4,144 women to the local government health facility, including 5,783/5,847 (99%) of those reporting that they had never previously registered for HIV care.

**Fig 3:**
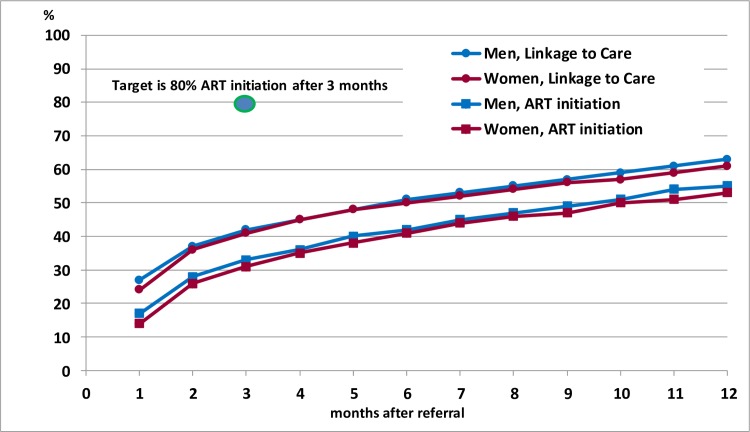
Time from referral to linkage to care and ART initiation during the first annual round of the PopART intervention. Survival curves showing cumulative proportions linking to care or initiating ART following referral by community HIV-care providers. ART, antiretroviral therapy.

Among the total of 6,197 adults who were not on ART at the time of the first household visit and were referred to HIV care, 42% (95% CI: 40%–43%) had initiated ART within 6 mo and 53% (95% CI: 52%–55%) within 12 mo, with very similar rates in men and women. Fig 3 shows survival plots for time from referral to linkage to care and ART initiation and indicates that by 3 mo only 41% (95% CI: 40%–42%) had linked to care and 32% (95% CI: 31%–33%) had initiated ART, but with additional individuals linking to care and starting ART each month up to at least 1 y.

### Change in knowledge of HIV status and linkage to care following intervention

At the time of the CHiP visit, 18% of men and 26% of women who consented to the intervention self-reported that they were HIV-positive or that they had tested HIV-negative in the previous 3 mo and thus knew their current HIV test status (Table 1). After the CHiP visit, as a result of the HIV tests provided by the CHiPs, the proportion who knew their current test status increased to 80% and 85%, respectively.

**Table 1:** Change in knowledge of HIV status, linkage to care, and ART uptake following the intervention among men and women.

Indicator	Before or after Round 1 CHiP visit	Men		Women	
		*n/N*	Percent	*n/N*	Percent
Knows HIV status/all who consented	Before^1^	8,305/45,399	18%	14,558/55,703	26%
	After^2^	36,398/45,399	80%	47,089/55,703	85%
Known HIV-positive/all who consented	Before^1^	2,423/45,399	5%	5,309/55,703	10%
	After^2^	4,138/45,399	9%	8,702/55,703	16%
In HIV care/all known HIV-positive	Before^2^	2,133/4,138	52%	4,765/8,702	55%
	After^3^	2,506/3,222	78%	5,528/7,043	78%
On ART/all known HIV-positive	Before^2^	1,941/4,138	47%	4,308/8,702	49%
	After^3^	2,309/3,222	72%	5,106/7,043	72%

^1^Prior to annual round visit.

^2^Immediately following annual round visit.

^3^At end of Round 1 (restricted to adults still resident in the same zone of the community at end of Round 1 according to last recorded information from Round 1).

ART, antiretroviral therapy; CHiP, community HIV-care provider.

Of the 4,138 men and 8,702 women known to be HIV-positive after the CHiP visit, 52% of men and 55% of women were already in HIV care, while 47% of men and 49% of women were already on ART. At the end of the first annual round, 3,222/4,138 (78%) of the known HIV-positive men and 7,043/8,702 (81%) of the known HIV-positive women were still resident in the same zone of the community according to the last available information. By the end of the first annual round of the intervention, the proportions of both men and women in care and on ART had increased to 78% and 72%, respectively.

### Reaching the 90-90-90 targets

Data on knowledge of HIV-positive status and linkage to care were used to estimate coverage against the first two of the 90-90-90 targets at three time points, before and immediately after the CHiP visit and at the end of the first annual round of the intervention (Tables 2 and 3). These estimates were obtained based on the assumptions described in the Methods.

**Table 2:** Estimated proportions of HIV+ individuals who knew their status and who were on ART before the CHiP visit, immediately after the CHiP visit, and at the end of Round 1, in those consenting to the intervention.

Indicator	Before CHiP visit	Immediately after CHiP visit	End Round 1^1^
**Men**			
Number HIV+ among those who consented to participate in Round 1 (estimated)	4,662	4,662	3,630
Number (percent) who know HIV+ status	2,423 (52%)	4,138 (89%)	3,222 (89%)
Number (percent) on ART	1,941 (80%)	1,941 (47%)	2,309 (72%)
Overall proportion on ART	42%	42%	64%
**Women**			
Number HIV+ among those who consented to participate in Round 1 (estimated)	9,499	9,499	7,688
Number (percent) who know HIV+ status	5,309 (56%)	8,702 (92%)	7,043 (92%)
Number (percent) on ART	4,308 (81%)	4,308 (49%)	5,106 (72%)
Overall proportion on ART	45%	45%	66%

^1^End Round 1 numbers and percentages are restricted to adults still resident in the same zone of the community at the end of Round 1, according to the most recently available information.

ART, antiretroviral therapy; CHiP, community HIV-care provider.

**Table 3:** Estimated proportions of HIV+ individuals who knew their status and who were on ART before the CHiP visit, immediately after the CHiP visit, and at the end of Round 1, in total adult population.

Indicator	Before CHiP visit	Immediately after CHiP visit	End Round 1^1^
**Men**			
Number HIV+ among total adult population in Round 1 (estimated)	6,649	6,649	5,206
Number (percent) who know HIV+ status	3,451 (52%)	5,166 (78%)	4,045 (78%)
Number (percent) on ART	2,769 (80%)	2,769 (54%)	2,975 (74%)
Overall proportion on ART	42%	42%	57%
**Women**			
Number HIV+ among total adult population in Round 1 (estimated)	11,037	11,037	8,958
Number (percent) who know HIV+ status	6,197 (56%)	9,590 (87%)	7,784 (87%)
Number (percent) on ART	5,036 (81%)	5,036 (53%)	5,715 (73%)
Overall proportion on ART	46%	46%	64%

^1^End Round 1 numbers and percentages are restricted to adults still resident in the same zone of the community at the end of Round 1, according to the most recently available information.

ART, antiretroviral therapy; CHiP, community HIV-care provider.

**Fig 4:**
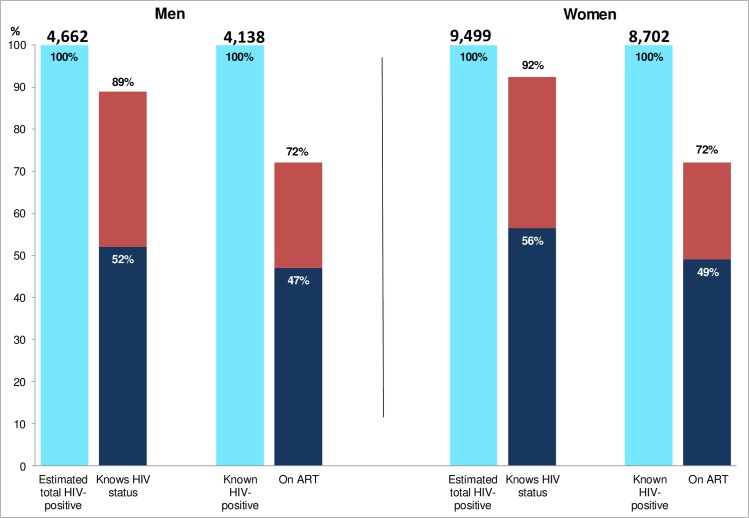
Estimated coverage compared with the first two 90 targets in those consenting to the PopART intervention. Dark blue bars show the estimated proportion of HIV+ adults who knew their status (first 90 target) and the estimated proportion of those who knew their HIV+ status who were on ART (second 90 target), pre-intervention. Red bars show the same estimated proportions, post-intervention. ART, antiretroviral therapy.

Among those consenting to the intervention, we estimate that prior to the intervention 52% of all HIV-positive men and 56% of all HIV-positive women knew their HIV-positive status. After the first annual round, these proportions increased to 89% of men and 92% of women, close to or exceeding the first 90 target (Fig 4).

Among those known HIV-positive prior to the CHiP visit, 80% of men and 81% of women reported that they were currently on ART. Immediately after the CHiP visit, this proportion decreased to 47% and 49% as a result of the increase in the denominator due to the large number of HIV-positive individuals newly diagnosed by the CHiPs. By the end of the annual round, however, the proportion on ART had increased to 72% and 72% in men and women, respectively.

**Fig 5:**
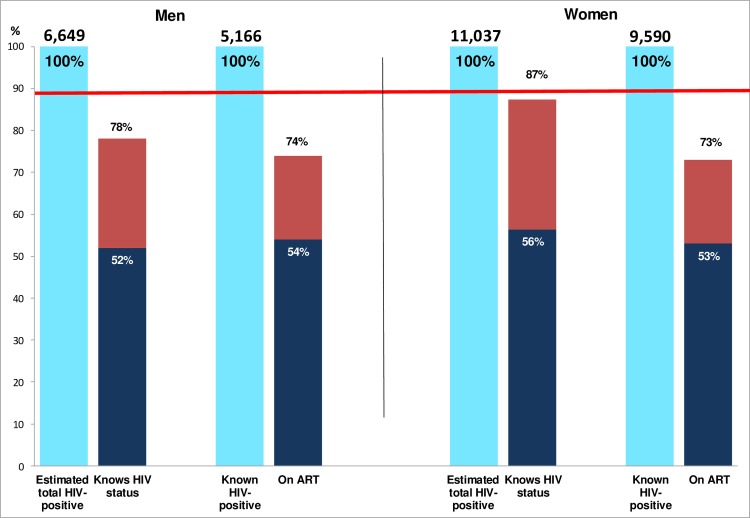
Estimated coverage compared with the first two 90 targets extrapolated to the total adult population. The red line shows the 90% target for first two of the 90-90-90 targets. Dark blue bars show the estimated proportion of HIV+ adults who knew their status (first 90 target) and the estimated proportion of those who knew their HIV+ status who were on ART (second 90 target), pre-intervention. Red bars show the same estimated proportions, post-intervention. ART, antiretroviral therapy.

Estimated coverage against the first two 90 targets in the entire adult population was somewhat lower because not everyone was enumerated or consented to the intervention. Based on the stated assumptions, we estimate that the proportion of HIV-positive individuals who knew their HIV-positive status increased from 52% of men and 56% of women before the intervention to 78% and 87%, respectively, after the first annual round. The proportion of known HIV-positive individuals on ART increased from 54% of men and 53% of women immediately after the CHiP visit to 74% and 73%, respectively, by the end of the annual round (Fig 5).

Table 2 also shows that the estimated overall proportion of HIV-positive adults on ART increased from 42% to 57% in men and from 46% to 64% in women, compared with the target of 81% based on the product of the first two of the 90-90-90 targets. S1 Fig shows an alternative display of coverage against the targets, similar to that used to report the Agence Nationale de Recherche sur le Sida et les Hépatites Virales 12249 Treatment as Prevention trial (ANRS 12249 TasP trial) [18].

**Fig 6:**
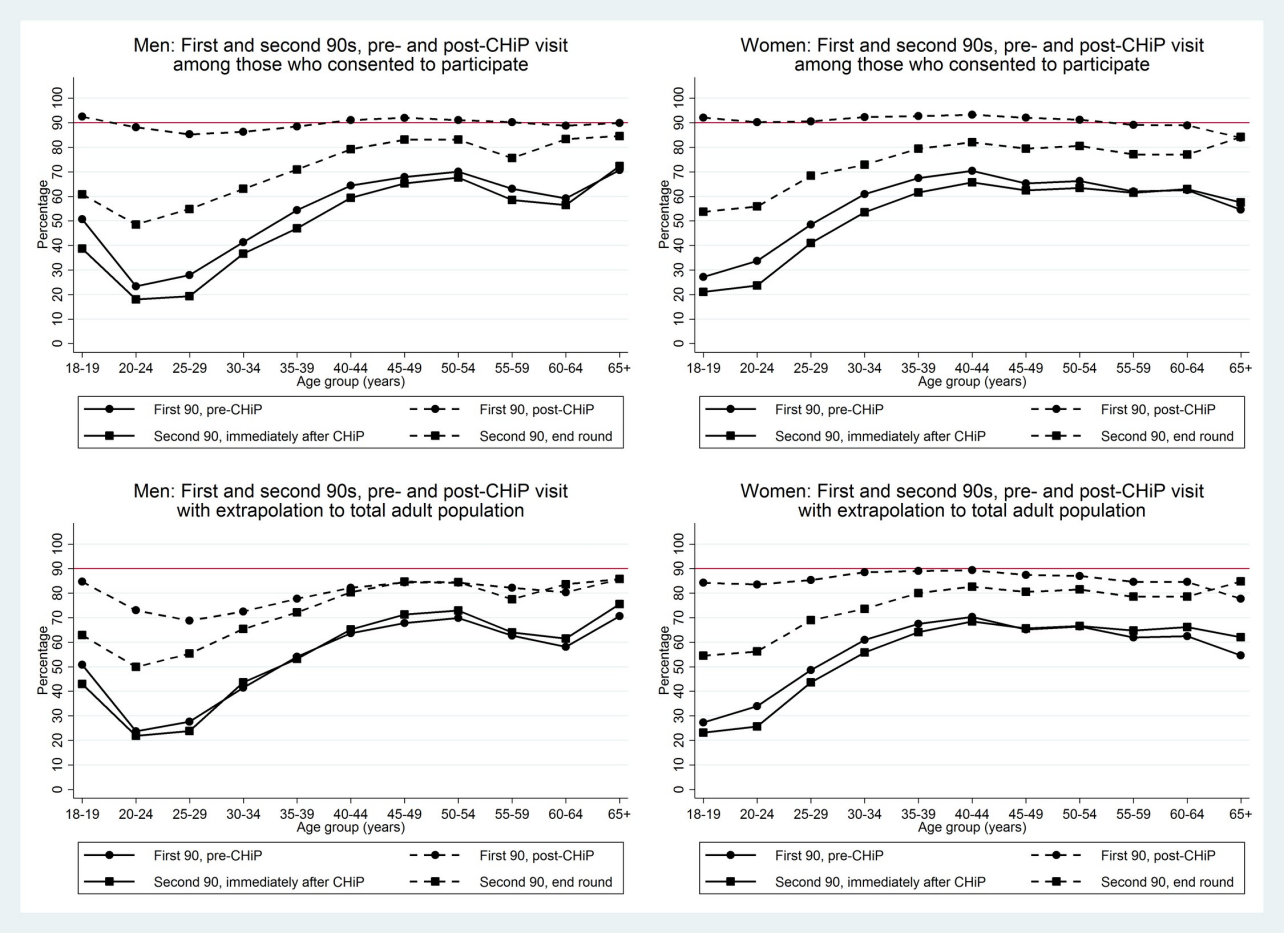
First two 90 target estimates by sex and age group in those consenting to the PopART intervention and extrapolated to the total adult population. The red line shows the 90% target for first two of the 90-90-90 targets. The first 90 target is proportion of individuals with knowledge of HIV-positive status, and the second 90 target is proportion of known HIV-positive individuals on antiretroviral therapy. CHiP, community HIV-care provider.

**Fig 7:**
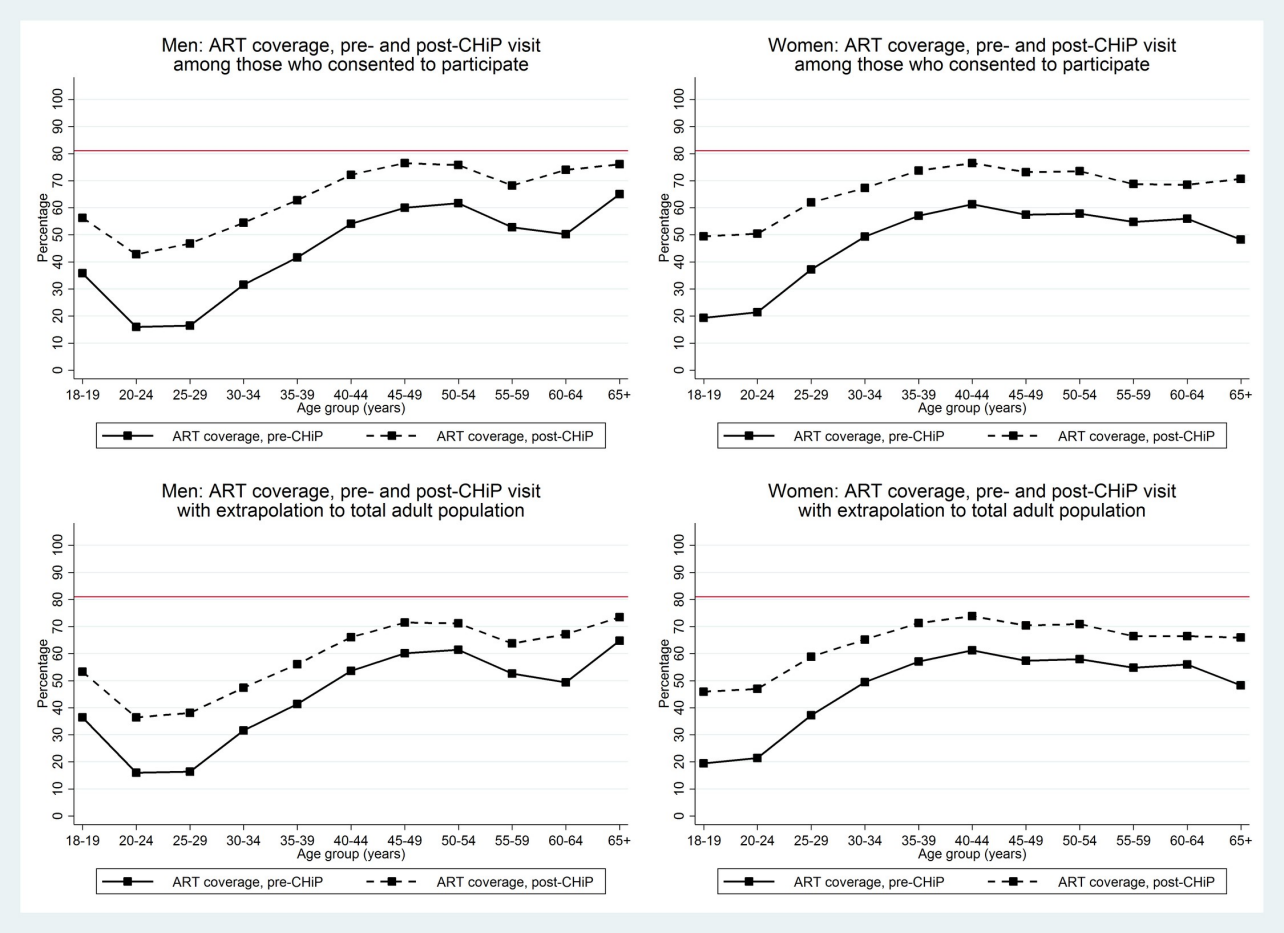
Overall estimates of proportion of HIV+ adults on ART by age group and sex in those consenting to the PopART intervention and extrapolated to the total adult population. The red line shows the cumulative 81% target based on first two of the 90-90-90 targets. ART, antiretroviral therapy; CHiP, community HIV-care provider.

Fig 6 shows the coverage estimates for the first and second 90 targets broken down by age group as well as sex. Prior to the CHiP intervention, knowledge of HIV-positive status and ART coverage were considerably lower in young men and women than in older age groups. HIV test uptake by young adults who participated in the intervention was high, so that by the end of the first annual round, knowledge of HIV-positive status was close to 90% in all age groups. In the entire population, estimated knowledge of HIV-positive status remained high among women, but fell to around 70%–80% among young men because of the relatively large number who could not be contacted by CHiPs during household visits (17%, 20%, 24%, and 26% of men aged 18–19, 20–24, 25–29, and 30–34 y, respectively, were not contacted by CHiPs during Round 1). In both men and women, by the end of the first annual round, ART coverage among known HIV-positive individuals was relatively high in older age groups, at approximately 80% among men ≥40 y and women ≥35 y, but remained lower (around 50%–60%) in the youngest men and women. Fig 7 shows overall estimates of the proportion of HIV-positive adults on ART by age group and sex. After the first annual round, these estimates were approaching the 81% target in older adults, but were considerably lower in young men and women.

### Sensitivity analysis

S5 Table shows two sensitivity analyses for the estimates of coverage for the first and second 90 targets. Focusing on coverage after the first annual round of the intervention, estimates were not sensitive to assumptions about adults who consented to the intervention. The sensitivity analyses produced a wider range of estimates when extrapolating to the entire adult population, although qualitative conclusions were broadly similar, with higher knowledge of HIV-positive status in women (82%–91%) than men (70%–84%) and with ART coverage among known HIV-positive individuals between 69%–79% in both men and women.

## Discussion

This study showed substantial increases in knowledge of HIV status and ART coverage after the first annual round of a UTT intervention delivered through a home-based service linked to government health facilities providing care and treatment for HIV. Among those consenting to the intervention, knowledge of current HIV status increased from around 20% to greater than 80%, while ART coverage among known HIV-positive individuals increased from around 50% to 70%. In the entire adult population, the estimated proportion of HIV-positive individuals who knew their status (first 90 target) increased from 52% to 78% in men and from 56% to 87% in women, while the proportion of known HIV-positive individuals on ART (second 90 target) increased from 54% after the CHiP visit to 74% by the end of the round in men and from 53% to 73% in women. The estimated overall proportion of HIV-positive adults on ART, irrespective of whether they knew their HIV status, increased from 44% to 61%, compared with the 81% UNAIDS target for the first two 90 targets.

The concept of treatment as prevention has revitalised efforts to bring the HIV epidemic under effective control. The rationale for the concept is strong, and if high coverage can be achieved, the evidence suggests that the incidence of new infections should be reduced substantially. As a result, it now seems realistic to consider scenarios in which HIV prevalence and incidence are reduced in the long term to low levels, such that HIV/AIDS no longer constitutes a major public health problem.

These potential benefits will not be achieved, however, unless high rates of coverage can be achieved and sustained. There is increasing recognition that this will be challenging, both because of the many barriers to testing and care in settings with severe resource constraints and because high uptake requires the long series of steps in the cascade of care to be successfully negotiated [19–22]. The 90-90-90 targets, if they can be achieved, imply that only 73% of individuals living with HIV will be virally suppressed and therefore unlikely to transmit the virus to others [15]. If coverage is reduced to 80% at each step, the proportion virally suppressed falls to 51%. The problem is compounded because each of the 90-90-90 targets itself involves multiple steps. For example, the first 90 target requires that an individual is offered a test (at regular intervals), accepts the test, and receives the correct result. The second 90 target requires that the individual is effectively linked to care, and then attends repeated clinic visits until ART is prescribed and commenced. The third 90 target requires retention in care, high adherence to ART, and a regimen that is effective in achieving viral suppression in that individual. Seen in this light, the 90-90-90 targets will not be easy to achieve.

There have been relatively few attempts to estimate coverage against the targets on a large scale in resource-poor settings in sub-Saharan Africa [23–28]. We have used data from the largest ongoing programme to deliver UTT at the population level to provide such estimates. We find that after one annual round of the PopART intervention in four large communities in Zambia, we have come close to achieving the first 90 target in women and are approaching it in men. The second 90 target has proven more challenging, and although we have increased the estimated proportion of known HIV-infected individuals on ART from around 53% to 73%, the coverage needs to be increased. Data on the third 90 target, relating to retention on ART and viral suppression, will be obtained during the second annual round of the intervention.

Our findings agree with other studies in showing that a house-to-house service is able to achieve high rates of testing and knowledge of HIV status among community members [29]. Detailed results on test uptake will be reported elsewhere, but indicate that coverage of HIV testing is systematically higher among women than men. While men are only slightly more likely than women to decline a test when it is offered by the CHiPs, a significant challenge is that they are often not found at home during house-to-house visits. Reductions in the high incidence of HIV among women, particularly young women, will be limited unless their male partners are effectively tested, linked to care, and virally suppressed on ART [30]. Other studies have also found poorer coverage among men with home-based testing, and have suggested that additional testing strategies such as mobile or occupational testing facilities or self-testing may be needed to achieve high coverage in this group [31].

As in other programmes, the time taken for those diagnosed HIV-positive to link to care and commence ART has been longer than anticipated [19,32,33]. Our initial target was for ART to be commenced in 80% of individuals within 3 mo of referral. In practice, we found that slightly over 50% of both men and women commenced ART within 12 mo of referral. While the survival curves show that more and more individuals start ART over time, we need to speed up the rates of linkage and commencement of ART, and this was identified as a major focus of the CHiP intervention for the second annual round. Impact on HIV incidence will be limited unless HIV-positive individuals can be linked to care and initiated on ART rapidly, with lifelong retention and adherence to ART.

Of key importance were our findings on differential coverage across age groups. After the first annual round of intervention, knowledge of HIV status was high across all age groups in women and in older men, but was considerably lower among young men. Test uptake was high among young men who consented to the intervention, but a relatively large number of young men could not be contacted by CHiPs. ART coverage at the end of the first annual round among known HIV-positive individuals was considerably lower in young men and women than in older men and women, consistent with previous research [34]. Linkage to care and initiation of ART among those referred by CHiPs were slightly slower among younger adults, and far fewer young adults were already on ART at the start of the intervention, and this depressed coverage for the second 90 target. These findings emphasise the importance of contacting and engaging young men and women, and linking them to care as rapidly as possible. Full data on uptake by age and sex will be published separately.

Three other community-randomised trials in sub-Saharan Africa are measuring the impact of UTT interventions on HIV incidence: the ANRS 12249 TasP trial in South Africa [18], the Sustainable East Africa Research on Community Health (SEARCH) trial in Kenya and Uganda [35], and the Botswana Combination Prevention Project (BCPP) trial in Botswana [26]. Results from the first of these, the ANRS 12249 TasP trial, have recently been reported [36]. In this study in KwaZulu-Natal, South Africa, six-monthly home-based testing resulted in high uptake of HIV testing among those contacted, and high rates of viral suppression 12 mo after ART initiation. As in our study, linkage to care and ART initiation (at clinics provided by the study) were slower than expected, with only 36% accessing care within 6 mo of referral. Analysis of HIV incidence showed no evidence of impact. Importantly, however, the home-based programme for HIV testing and linkage to care was delivered to both intervention and control arms of the study, which differed only in the threshold for ART initiation. Overall coverage of ART was similar in the two study arms, and so it is unsurprising that no impact on HIV incidence was observed. In contrast, the HPTN 071 (PopART) trial will compare HIV incidence in intervention communities receiving the full UTT intervention with that in control communities receiving current standard of care (not including home-based testing).

Our study has a number of limitations. Data on coverage are only collected on those who participate in the CHiP intervention, and in order to obtain estimates for the entire adult population, we had to make assumptions about those not participating. We also rely on self-report of known HIV-positive status and whether individuals are in care or on ART, although documentary evidence is sought where possible. Another limitation is that our estimates of coverage ignore any new HIV seroconversions occurring between the initial annual round visit and the end of the round, although these are likely to have only a small effect, given the average duration of follow-up and expected HIV incidence. A further limitation is that data were collected only on adults aged 18 y and over. Although these will encompass a large proportion of those with HIV infection, we know that there is a continuing high incidence and prevalence in young people in many settings. Following ethical review, we are now able to collect data on minors, subject to parental consent, and so in future will be able to document uptake in this important age group.

Strengths of our study are that we have collected data systematically for most households and a large proportion of adults in these large communities, and have stated our methods and assumptions clearly. We have also carried out analyses to examine the sensitivity of our estimates to the assumptions made. We believe that comparisons of coverage estimates between countries, settings, and programmes are susceptible to significant bias and distortion due to different availability of data and assumptions made in the analyses, and some harmonisation of such analyses is called for. The Universal Test and Treat Trials Consortium (UT^3^C), which comprises investigators from five randomised trials of UTT interventions in sub-Saharan Africa, is planning joint cross-study analyses to further explore this issue.

This paper reports uptake following the first year of the PopART intervention. A strength of this intervention is that household visits are made to all households during each annual round, as well as repeat visits during each year as appropriate. This approach offers the opportunity to continuously increase coverage, by accessing individuals who were previously not at home, offering tests to those who initially declined testing, and making special efforts to encourage linkage to care where this has not already occurred. After the first year, estimated overall coverage against the first two 90 targets combined has increased from 44% to 61%, indicating the gap still to be filled to reach the target of 81%. It will be important to document how uptake evolves over time, and we plan to report this after the second annual round of the intervention. The initial data, however, demonstrate that a community-wide intervention provided through a house-to-house service in communities with severe generalised HIV epidemics may be effective in achieving or approaching the 90-90-90 targets.

## Supporting information

S1 STROBE Checklist (DOC)

S1 Data Aggregate dataset. (XLSX)

S2 Data Definition of variables in aggregate dataset. (DOCX)

S1 Fig Estimated uptake in total adult population—alternative display. (PPTX)

S1 Table Estimate of the proportion of HIV+ women who knew their HIV+ status, among those aged 25–29 y who consented to participate in the CHiP intervention. (DOCX)

S2 Table Estimate of uptake of ART, among HIV+ women aged 25–29 y who consented to participate in the CHiP intervention and who knew their HIV+ status following the Round 1 annual household visit. (DOCX)

S3 Table Estimate of the proportion with knowledge of HIV+ status among all HIV+ women in the population aged 25–29 y (extrapolation to women who did not participate in the CHiP intervention in Round 1). (DOCX)

S4 Table Estimate of uptake of ART among all HIV+ women in the population aged 25–29 y who knew their HIV+ status (extrapolation to women who did not participate in the CHiP intervention in Round 1). (DOCX)

S5 Table Summary table of estimates for the first two 90-90-90 targets, Zambia Round 1; adults aged ≥18 y at time of annual round visit; sensitivity analysis. (DOCX)

## Abbreviations

ANRS 12249 TasP trial: Agence Nationale de Recherche sur le Sida et les Hépatites Virales 12249 Treatment as Prevention trial
ART: antiretroviral therapy
CHiP: community HIV-care provider
EDC: electronic data capture
TB: tuberculosis
UNAIDS: Joint United Nations Programme on HIV/AIDS
UTT: universal testing and treatment
