# Supplementary material for: A universal testing and treatment intervention to improve HIV control: One-year results from intervention communities in Zambia in the HPTN 071 (PopART) cluster-randomised trial
Source: PLoS Med. 2017 May 2;14(5):e1002292. doi: 10.1371/journal.pmed.1002292 (PMC5412988; doi:10.1371/journal.pmed.1002292)
Supplement: S1 STROBE Checklist — (DOC) [file pmed.1002292.s001.doc]

STROBE Statement—Checklist of items that should be included in reports of ***cohort studies***

|  | Item No | Recommendation |
| --- | --- | --- |
| **Title and abstract** | 1 | (*a*) Indicate the study’s design with a commonly used term in the title or the abstract **[Title describes study as “cluster-randomised trial”.]** |
|  |  | (*b*) Provide in the abstract an informative and balanced summary of what was done and what was found **[‘What was done’ is described in the first paragraph of the abstract section ‘Methods and findings’. ‘What was found’ is described in the abstract, ‘Methods and findings’ section, paragraph 2.]** |
| Introduction | | |
| Background/rationale | 2 | Explain the scientific background and rationale for the investigation being reported **[See ‘Introduction’ section, paragraphs 1-4.]** |
| Objectives | 3 | State specific objectives, including any prespecified hypotheses **[Primary objective of overall study and aims of the current analysis described in last paragraph (paragraph 5) of introduction.]** |
| Methods | | |
| Study design | 4 | Present key elements of study design early in the paper **[Provided in first two paragraphs of ‘Methods: Trial Design’.]** |
| Setting | 5 | Describe the setting, locations, and relevant dates, including periods of recruitment, exposure, follow-up, and data collection **[Provided in the three paragraphs of the ‘Methods: Intervention’ section.]** |
| Participants | 6 | (*a*) Give the eligibility criteria, and the sources and methods of selection of participants. Describe methods of follow-up **[Details provided in paragraph 2 of ‘Methods: Intervention’ section.]** |
|  |  | (*b*) For matched studies, give matching criteria and number of exposed and unexposed **[Not applicable]** |
| Variables | 7 | Clearly define all outcomes, exposures, predictors, potential confounders, and effect modifiers. Give diagnostic criteria, if applicable **[All measures defined in the ‘Methods: Statistical methods’ section.]** |
| Data sources/ measurement | 8* | For each variable of interest, give sources of data and details of methods of assessment (measurement). Describe comparability of assessment methods if there is more than one group. **[Source of data is provided in the section ‘Methods: Intervention’ paragraph 2 and methods of arriving at key variables is provided in the ‘Methods: Statistical Methods’ section. Methods of assessment of HIV endpoints provided in the ‘Methods: Laboratory methods’ section.]** |
| Bias | 9 | Describe any efforts to address potential sources of bias. **[Discussed in the ‘Methods: Statistical methods’ section paragraphs 4-7, supplementary tables 1-4, and sensitivity analysis in supplementary table 5.]** |
| Study size | 10 | Explain how the study size was arrived at **[Study size for the main trial is shown in Fig 1., and for referral is made to a previously-published paper on the study’s design (reference 17) where the rationale for the main study size is provided. Analyses in this manuscript were based upon the total population of study communities.]** |
| Quantitative variables | 11 | Explain how quantitative variables were handled in the analyses. If applicable, describe which groupings were chosen and why **[How quantitative variables were analysed covered in the ‘Methods: Statistical methods’ section.]** |
| Statistical methods | 12 | (*a*) Describe all statistical methods, including those used to control for confounding **[All statistical methods are described in the ‘Methods: Statistical methods’ section]** |
|  |  | (*b*) Describe any methods used to examine subgroups and interactions **[Not applicable]** |
|  |  | (*c*) Explain how missing data were addressed **[Described in the ‘Methods: Statistical methods’ section, paragraphs 3-7.]** |
|  |  | (*d*) If applicable, explain how loss to follow-up was addressed **[Not applicable]** |
|  |  | (*e*) Describe any sensitivity analyses **[Described in the last paragraph of the ‘Methods: Statistical Methods’ section, and in Supplementary Table 5.]** |
| Results | | |
| Participants | 13* | (a) Report numbers of individuals at each stage of study—eg numbers potentially eligible, examined for eligibility, confirmed eligible, included in the study, completing follow-up, and analysed **[Refer to the first paragraph of ‘Results: Cascade of care’ section and Fig 2.]** |
|  |  | (b) Give reasons for non-participation at each stage **[Not applicable]** |
|  |  | (c) Consider use of a flow diagram **[Fig 2]** |
| Descriptive data | 14* | (a) Give characteristics of study participants (eg demographic, clinical, social) and information on exposures and potential confounders **[Not applicable]** |
|  |  | (b) Indicate number of participants with missing data for each variable of interest **[Not applicable]** |
|  |  | (c) Summarise follow-up time (eg, average and total amount) **[Not applicable]** |
| Outcome data | 15* | Report numbers of outcome events or summary measures over time **[Data on the variables of interest are provided in the ‘Results’ section and figures.]** |
| Main results | 16 | (*a*) Give unadjusted estimates and, if applicable, confounder-adjusted estimates and their precision (eg, 95% confidence interval). Make clear which confounders were adjusted for and why they were included **[Results for the key variables of interest are provided throughout the ‘Results’ section text and tables.]** |
|  |  | (*b*) Report category boundaries when continuous variables were categorized **[Not applicable]** |
|  |  | (*c*) If relevant, consider translating estimates of relative risk into absolute risk for a meaningful time period **[Not applicable]** |
| Other analyses | 17 | Report other analyses done—eg analyses of subgroups and interactions, and sensitivity analyses **[Analysis by age and gender is provided in Figs 5 and 6. Sensitivity analysis is provided in S5 Table.]** |
| Discussion | | |
| Key results | 18 | Summarise key results with reference to study objectives **[‘Discussion’ section, especially the first paragraph.]** |
| Limitations | 19 | Discuss limitations of the study, taking into account sources of potential bias or imprecision. Discuss both direction and magnitude of any potential bias **[Paragraph on limitations—9^th^ paragraph in ‘Discussion’ section, p.11.]** |
| Interpretation | 20 | Give a cautious overall interpretation of results considering objectives, limitations, multiplicity of analyses, results from similar studies, and other relevant evidence **[Discussion’ section paragraphs 4-8]** |
| Generalisability | 21 | Discuss the generalisability (external validity) of the study results  **[In different sections of the ‘Discussion’ section, particularly paragraphs 1, 4-11.]** |
| Other information | | |
| Funding | 22 | Give the source of funding and the role of the funders for the present study and, if applicable, for the original study on which the present article is based **[This is provided in the ‘Finance statement’ within the metadata.]** |

*Give information separately for exposed and unexposed groups.

**Note:** An Explanation and Elaboration article discusses each checklist item and gives methodological background and published examples of transparent reporting. The STROBE checklist is best used in conjunction with this article (freely available on the Web sites of PLoS Medicine at http://www.plosmedicine.org/, Annals of Internal Medicine at http://www.annals.org/, and Epidemiology at http://www.epidem.com/). Information on the STROBE Initiative is available at http://www.strobe-statement.org.
