# Supplementary material for: A universal testing and treatment intervention to improve HIV control: One-year results from intervention communities in Zambia in the HPTN 071 (PopART) cluster-randomised trial
Source: PLoS Med. 2017 May 2;14(5):e1002292. doi: 10.1371/journal.pmed.1002292 (PMC5412988; doi:10.1371/journal.pmed.1002292)
Supplement: S2 Data — (DOCX) [file pmed.1002292.s003.docx]

**Definition of variables in aggregated dataset**

| Variable name | Variable description | Coding |
| --- | --- | --- |
| gender | Gender | 1= Male; 2=Female |
| community | Community of residence | 1,2,3,4 |
| agegroup | Age group (years) | 1=18-19; 2=20-24; 3=25-29 4=30-34; 5=35-39; 6=40-44; 7=45-49; 8=50-54; 9=55-59; 10=60-64; 11=65+ |
| prop_hh_enumerated | Proportion of households that consented to enumeration, among visited households |  |
| enumerated | Enumerated as a household member | Count of individuals |
| consented | Consented to participate in intervention | Count of individuals |
| self_report_hivpos | Self-reported HIV-positive | Count of individuals |
| tested_for_hiv | Tested for HIV, among those who did not self-report HIV-positive | Count of individuals |
| tested_hiv_negative_3M | Did not self-report HIV-positive, and did not accept offer of HIV testing, but self-reported an HIV-negative test result in the 3 months prior to the Round 1 household visit | Count of individuals |
| know_hivstatus | Knows HIV status immediately after Round 1 household visit | Count of individuals |
| tested_hivpos | Tested HIV-positive | Count of individuals |
| known_hivpos | Known HIV-positive (self-reported HIV-positive plus tested HIV-positive) | Count of individuals |
| on_art_baseline | Self-reported on ART at time of Round 1 household visit | Count of individuals |
| known_hivpos_resident_end_round1 | Resident in the same area of the community at the end of Round 1 according to last information collected in Round 1, among individuals who were known to be HIV-positive | Count of individuals |
| on_art_end_round1 | On ART at the end of Round 1, among those still resident in the same area of the community according to the last information collected in Round 1 | Count of individuals |
