# Supplementary figures and images for: A universal testing and treatment intervention to improve HIV control: One-year results from intervention communities in Zambia in the HPTN 071 (PopART) cluster-randomised trial
Source: PLoS Med. 2017 May 2;14(5):e1002292. doi: 10.1371/journal.pmed.1002292 (PMC5412988; doi:10.1371/journal.pmed.1002292)

## Slide 1
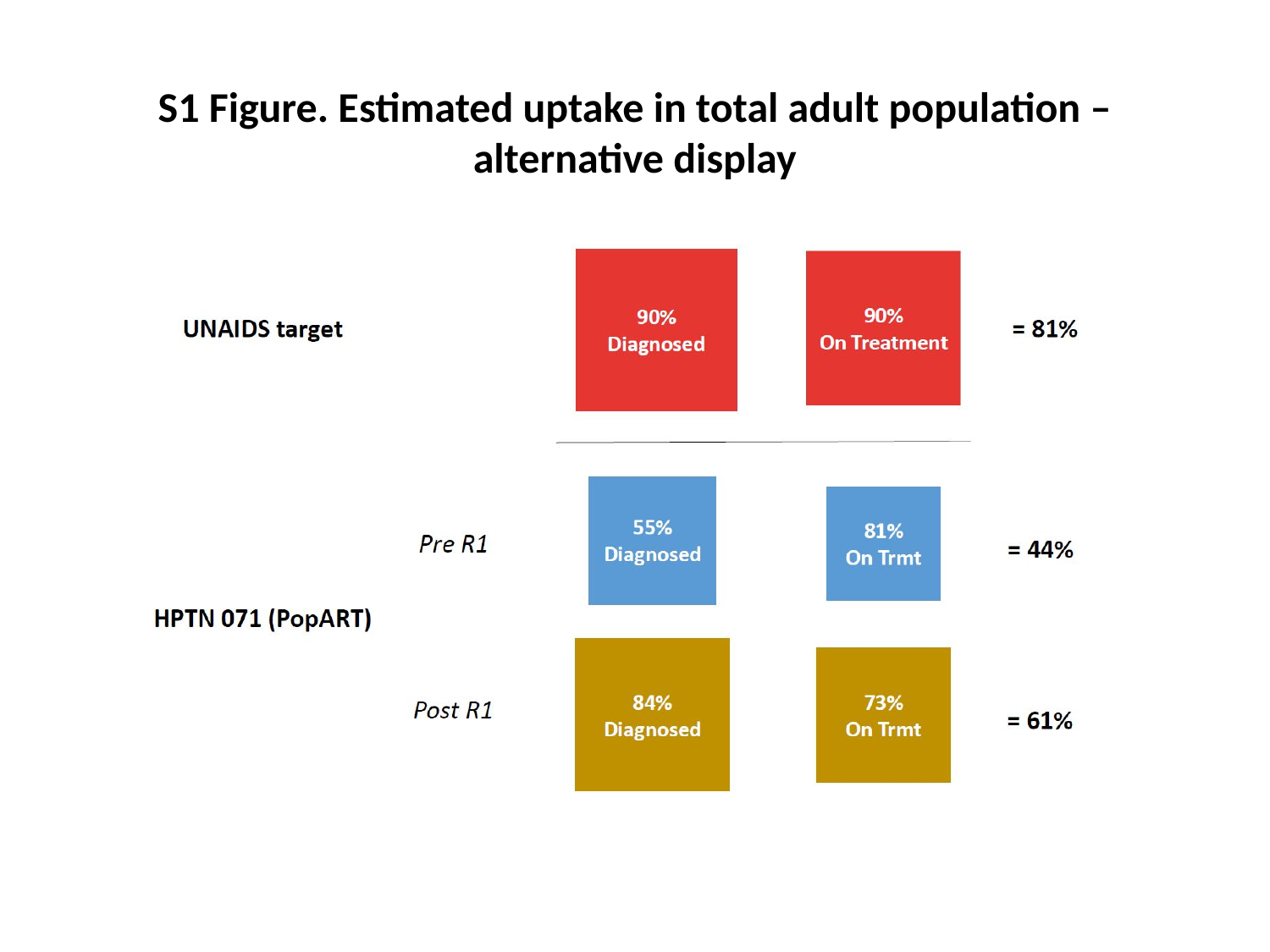

# S1 Figure. Estimated uptake in total adult population – alternative display

Supplement: S1 Fig — (PPTX) [file pmed.1002292.s004.pptx]
