# Supplementary material for: A universal testing and treatment intervention to improve HIV control: One-year results from intervention communities in Zambia in the HPTN 071 (PopART) cluster-randomised trial
Source: PLoS Med. 2017 May 2;14(5):e1002292. doi: 10.1371/journal.pmed.1002292 (PMC5412988; doi:10.1371/journal.pmed.1002292)
Supplement: S1 Table — (DOCX) [file pmed.1002292.s005.docx]

S1 Table. Estimate of the proportion of HIV+ women who knew their HIV+ status, among those aged 25-29 years who consented to participate in the CHiP intervention

|  | Number consented to participate | Proportion whose HIV status is not known to the CHiPs^1^ | Number who self-reported HIV+ | Number who self-reported or tested HIV+, i.e. total “known HIV+” following CHiPs visit | HIV prevalence, among adults who did not self-report HIV+ and accepted HCT | Estimated number of HIV+ adults, among those whose HIV status is not known to the CHiPs^2^ | Estimated total HIV+ adults, among those who consented to participate^3^ |
| --- | --- | --- | --- | --- | --- | --- | --- |
| Column identifier: | A | B | C | D | E | F | G |
| Community |  |  |  |  |  |  |  |
| 1 | 1233 | 0.295 | 69 | 127 | 0.083 | 30.3 | 157.3 |
| 2 | 2008 | 0.119 | 174 | 372 | 0.136 | 32.6 | 404.6 |
| 3 | 5022 | 0.111 | 395 | 787 | 0.115 | 63.8 | 850.8 |
| 4 | 1946 | 0.192 | 260 | 390 | 0.128 | 47.7 | 437.7 |
| **Total** | **10209** |  | **898** | **1676** |  | **174.4** | **1850.4** |
| **Knowledge of HIV+ status** |  |  | **48.5% (898/1850.4)** | **90.6% (1676/1850.4)** |  |  |  |

^1^ An individual’s HIV status is known to the CHiPs if they self-reported HIV+, or accepted HIV testing, or self-reported an HIV-negative test result in the previous 3 months; ^2^ Calculated as: Number who consented to participate x proportion whose HIV status is not known to the CHiPs x HIV prevalence among adults who accepted HCT, i.e. Column A x Column B x Column E; ^3^ Calculated as the number who self-reported HIV+ or tested HIV+ i.e. total who were known to the CHiPs as HIV+ following the household visit (Column D), plus the estimated number of HIV+ adults among those whose HIV status is not known to the CHiPs (Column F).
