# Supplementary material for: A universal testing and treatment intervention to improve HIV control: One-year results from intervention communities in Zambia in the HPTN 071 (PopART) cluster-randomised trial
Source: PLoS Med. 2017 May 2;14(5):e1002292. doi: 10.1371/journal.pmed.1002292 (PMC5412988; doi:10.1371/journal.pmed.1002292)
Supplement: S2 Table — (DOCX) [file pmed.1002292.s006.docx]

S2 Table. Estimates of uptake of ART, among HIV+ women aged 25-29 years who consented to participate in the CHiP intervention and who knew their HIV+ status following the Round 1 annual household visit

|  | Number who self-reported or tested HIV+, i.e. total “known HIV+” following CHiPs visit | Number on ART at time of Round 1 household visit | Proportion on ART at time of Round 1 household visit | Number resident at end of Round 1 | Number on ART at end of Round 1, among those still resident end of Round 1 | Proportion on ART at end of Round 1, among those still resident end of Round 1 | Proportion resident at end of Round 1 |
| --- | --- | --- | --- | --- | --- | --- | --- |
| Column identifier: | A | B | C | D | E | F | G |
| Community |  |  |  |  |  |  |  |
| 1 | 127 | 48 | 0.378 | 106 | 78 | 0.736 | 0.835 |
| 2 | 372 | 136 | 0.366 | 264 | 190 | 0.720 | 0.710 |
| 3 | 787 | 308 | 0.391 | 596 | 382 | 0.641 | 0.757 |
| 4 | 390 | 196 | 0.503 | 335 | 240 | 0.716 | 0.859 |
| **Total** | **1676** | **688** |  | **1301** | **890** |  |  |
| **Uptake of ART, among HIV+ adults who know their HIV+ status** |  | **41.1%**  **(688/1676)** |  |  | **68.4%**  **(890/1301)** |  |  |
