# Supplementary material for: A universal testing and treatment intervention to improve HIV control: One-year results from intervention communities in Zambia in the HPTN 071 (PopART) cluster-randomised trial
Source: PLoS Med. 2017 May 2;14(5):e1002292. doi: 10.1371/journal.pmed.1002292 (PMC5412988; doi:10.1371/journal.pmed.1002292)
Supplement: S3 Table — (DOCX) [file pmed.1002292.s007.docx]

S3 Table. Estimate of knowledge of HIV+ status among all HIV+ women in the population aged 25-29 years (extrapolation to women who did not participate in the CHiP intervention in Round 1)

|  | Number enumerated | Proportion of households enumerated | Estimated total population^1^ | Estimated number who did not participate in intervention^2^ | Estimated number of HIV+ individuals, among those who did not participate^3^ | Estimated proportion of HIV+ individuals who know their HIV+ status, among those who did not participate^4^ | Estimated number of HIV+ individuals who know their HIV+ status, among those who did not participate^5^ | Estimated total HIV+ population^6^ | Estimated number of HIV+ individuals who know their HIV+ status, following annual round visit^7^ |
| --- | --- | --- | --- | --- | --- | --- | --- | --- | --- |
| Column identifier: | A | B | C | D | E | F | G | H | I |
| Community |  |  |  |  |  |  |  |  |  |
| 1 | 1338 | 0.970 | 1379.4 | 146.4 | 18.7 | 0.439 | 8.2 | 176.0 | 135.2 |
| 2 | 2247 | 0.986 | 2278.9 | 270.9 | 54.6 | 0.430 | 23.5 | 459.2 | 395.5 |
| 3 | 5489 | 0.960 | 5717.7 | 695.7 | 117.9 | 0.464 | 54.7 | 968.7 | 841.7 |
| 4 | 2120 | 0.929 | 2282.0 | 336.0 | 75.6 | 0.594 | 44.9 | 513.3 | 434.9 |
| **Total** | **11194** |  | **11658.0** | **1449.0** | **266.7** |  | **131.3** | **2117.1** | **1807.3** |
| **Knowledge of HIV+ status before CHiP intervention** |  |  |  |  |  |  |  |  | **48.6%**  **(1029.3/2117.1)^8^** |
| **Knowledge of HIV+ status post-CHiP intervention** |  |  |  |  |  |  |  |  | **85.4%**  **(1807.3/2117.1)** |

^1^ Estimated as Column A / Column B; ^2^ Calculated as Column C – number who consented to participate (Column A of Table 1); ^3^ Calculated as (Column G of Table 1 / Column A of Table 1) x Column D; ^4^ Estimated as (Column C of Table 1 / Column G of Table 1); ^5^ Estimated as Column E x Column F; ^6^ Estimated as Column G of Table 1 + Column E; ^7^ Estimated as Column D of Table 1 + Column G; ^8^ Numerator calculated as Column C of Table 1 + Column G
