# Supplementary material for: A universal testing and treatment intervention to improve HIV control: One-year results from intervention communities in Zambia in the HPTN 071 (PopART) cluster-randomised trial
Source: PLoS Med. 2017 May 2;14(5):e1002292. doi: 10.1371/journal.pmed.1002292 (PMC5412988; doi:10.1371/journal.pmed.1002292)
Supplement: S4 Table — (DOCX) [file pmed.1002292.s008.docx]

S4 Table. Estimate of uptake of ART among all HIV+ women in the population aged 25-29 years who knew their HIV+ status (extrapolation to women who did not participate in the CHiP intervention in Round 1)

|  | Proportion on ART at time of Round 1 household visit, among individuals who participated in the intervention and self-reported HIV+^1^ | Estimated number of HIV+ individuals who know their HIV+ status, among those who did not participate^2^ | Estimated number on ART at time of Round 1 household visit, among HIV+ individuals who did not participate in the intervention but know their HIV+ status^3^ | Total who know their HIV+ status, following Round 1 household visit^4^ | Total on ART at time of Round 1 visit, among those who know their HIV+ status following Round 1 household visit^5^ | Proportion resident at end of Round 1, among those who knew their HIV+ status and did not consent to participate^6^ | Estimated number resident at end of Round 1, among those who knew their HIV+ status and did not consent to participate^7^ | Estimated number on ART at end of Round 1, among those who did not consent to participate^8^ | Total on ART by end of Round 1, among those who know their HIV+ status following Round 1 household visit and are still resident^9^ |
| --- | --- | --- | --- | --- | --- | --- | --- | --- | --- |
| Column identifier: | A | B | C | D | E | F | G | H | I |
| Community |  |  |  |  |  |  |  |  |  |
| 1 | 0.696 | 8.2 | 5.7 | 135.2 | 53.7 | 0.835 | 6.8 | 4.8 | 82.8 |
| 2 | 0.782 | 23.5 | 18.3 | 395.5 | 154.3 | 0.710 | 16.7 | 13.0 | 203.0 |
| 3 | 0.780 | 54.7 | 42.7 | 841.7 | 350.7 | 0.757 | 41.4 | 32.3 | 414.3 |
| 4 | 0.754 | 44.9 | 33.8 | 434.9 | 229.8 | 0.859 | 38.6 | 29.1 | 269.1 |
| **Total** |  | **131.3** | **100.6** | **1807.3** | **788.6** |  | **103.5** | **79.2** | **969.2** |
| **Uptake of ART, among HIV+ adults who know their HIV+ status** |  |  |  |  | **43.6%**  **(788.6/1807.3)** |  |  |  | **69.0%**  **(969.2/1404.5)^10^** |

^1^ Calculated as Column B of Table 2 / Column C of Table 1; ^2^ Column G of Table 3; ^3^ Calculated as Column A x Column B; ^4^ Column I of Table 3; ^5^ Calculated as Column C + Column B of Table 2; ^6^ Assumed to be the same as for HIV+ adults who consented to participate and knew their HIV+ status following the Round 1 household visit, i.e. Column G of Table 2; ^7^ Calculated as Column F x Column B; ^8^ Calculated as Column F x Column C; ^9^ Calculated as Column H + Column E of Table 2; ^10^ Denominator is calculated as 103.5 + 1301, i.e. Column G total + Column D total of Table 2
