# Supplementary material for: A universal testing and treatment intervention to improve HIV control: One-year results from intervention communities in Zambia in the HPTN 071 (PopART) cluster-randomised trial
Source: PLoS Med. 2017 May 2;14(5):e1002292. doi: 10.1371/journal.pmed.1002292 (PMC5412988; doi:10.1371/journal.pmed.1002292)
Supplement: S5 Table — (DOCX) [file pmed.1002292.s009.docx]

S5 Table. Summary table of estimates for the first two 90-90-90 targets, Zambia Round 1; adults age ≥18 years at time of annual round visit; sensitivity analysis

|  | **Men** | | | | | **Women** | | | | |
| --- | --- | --- | --- | --- | --- | --- | --- | --- | --- | --- |
|  |  | **First 90 (%)** | | **Second 90 (%)** | |  | **First 90 (%)** | | **Second 90 (%)** | |
|  |  | **Pre-CHiP** | **Immediately after annual round visit**  **(Post-CHiP)** | **Immediately after annual round visit** | **End round** |  | **Pre-CHiP** | **Immediately after annual round visit**  **(Post-CHiP)** | **Immediately after annual round visit** | **End round** |
|  | **N^1^** |  |  |  |  | **N^1^** |  |  |  |  |
| Consented to participate | **4662** | **52** | **89** | **47** | **72** | **9499** | **56** | **92** | **49** | **72** |
| *Range in sensitivity analysis (1)^2^* | ***4557-4793*** | ***51-53*** | ***86-91*** | ***47*** | ***72*** | ***9340-9699*** | ***55-57*** | ***90-93*** | ***49*** | ***72*** |
| *Range in sensitivity analysis (2)^3^* | ***5186*** | ***58*** | ***91*** | ***46-51*** | ***67-73*** | ***10297*** | ***61*** | ***94*** | ***49-53*** | ***69-73*** |
| Extrapolated to total population | **6649** | **52** | **78** | **54** | **74** | **11037** | **56** | **87** | **53** | **73** |
| *Range in sensitivity analysis (1)^2^* | ***6112-7345*** | ***47-58*** | ***70-84*** | ***49-61*** | ***70-79*** | ***10549-11662*** | ***53-60*** | ***82-91*** | ***50-56*** | ***72-76*** |
| *Range in sensitivity analysis (2)^3^* | ***7390*** | ***58*** | ***81*** | ***52-57*** | ***69-74*** | ***11965*** | ***61*** | ***90*** | ***51-55*** | ***70-74*** |

^1^ Estimated number of HIV-positive adults in total adult population, with extrapolation for (1) adults who participated but whose HIV status is not known to the CHiPs (2) adults who did not participate

*^2^ Sensitivity analysis (1) across 4 key assumptions: (A) HIV prevalence among adults who participated but their HIV status is not known to CHiPs. Central estimate = HIV prevalence among those who participated and accepted HIV testing; (B) HIV prevalence among adults who did not participate. Central estimate = HIV prevalence among those who participated (C) Percentage of HIV+ adults who know their HIV+ status, among HIV+ adults who did not participate. Central estimate = Percentage of HIV+ adults who self-reported they were HIV-positive at annual round visit, among those who participated (D) Percentage of HIV+ adults who are on ART, among HIV+ adults who know their HIV+ status and did not participate. Central estimate = Percentage of HIV+ adults who were on ART at time of annual round visit, among those who participated and self-reported they were HIV-positive. All of (A)-(D) are varied in a range 1.25 times lower or higher than the central estimate. In the supplementary tables, the sensitivity analysis is applied as follows: (A) to Table 1 column F; (B) to Table 3 Column E; (C) to Table 3 Column F; and (D) to Table 4 Column A*

*^3^ Sensitivity analysis (2): Among adults who participated but their HIV status is not known to CHiPs, HIV prevalence is assumed to be twice the value observed among adults who accepted the offer of HIV testing from CHiPs. Among adults whose HIV status is not known to CHiPs, and are HIV-positive, the percentage who know their HIV+ status is assumed to be the same as for adults whose HIV status is known to CHiPs i.e. equal to (self-reported HIV+ to CHiPs / total known by the CHiPs to be HIV+). Among adults who know their HIV+ status, two alternative assumptions are made for the percentage who were on ART at the time of the annual round visit (a) the percentage on ART is the same as for adults who self-reported to CHiPs that they were HIV+ (b) the percentage on ART is half the value for adults who self-reported to CHiPs that they were HIV+*
